# Carbon Dots Derived from Carbonized Citri Reticulatae Pericarpium (Chenpi) Alleviate Metabolic Dysfunction-Associated Steatohepatitis

**DOI:** 10.3390/molecules31213888

**Published:** 2026-10-28

**Authors:** Juan Luo, Xiangling Chen, Ping Zhang, Yuanchao Shi, Yanling Li, Peng Cui, Qiongye Dong

**Affiliations:** 1 Precision Medicine Institute, Peking University Shenzhen Hospital, Shenzhen 518036, China; 2 Department of Hematology, Peking University Shenzhen Hospital, Shenzhen 518036, China; 3 Central Laboratory, Peking University Shenzhen Hospital, Shenzhen 518036, China; 4 Intervention and Cell Therapy Center, Peking University Shenzhen Hospital, Shenzhen 518036, China

**Keywords:** *Citri Reticulatae Pericarpium*, carbon dots, MASH, carbonized

## Abstract

Metabolic dysfunction-associated steatohepatitis (MASH) prevalence is rising globally, with approved targeted therapies associated with substantial adverse reactions. In this study, we synthesized carbon dots (CDs) derived from *Citri Reticulatae Pericarpium* (CRP-CDs) to investigate their safety and therapeutic effect against MASH. Here, novel CRP-CDs were synthesized via high-temperature carbonization and systematically characterized in terms of particle size, surface functional groups, and optical properties. We assessed the in vitro and in vivo biosafety of CRP-CDs. An FFA-induced fatty-degeneration HepG2 cell model was used to examine its regulation of hepatic lipid accumulation, while the HFD-induced mouse model was employed to evaluate its therapeutic efficacy against MASH. Data-independent acquisition mass spectrometry (DIA-MS) quantitative proteomics was performed to decipher its anti-MASH molecular mechanisms. The results showed that CRP-CDs displayed uniform particle size, abundant surface functional groups, and stable optical behaviors based on characterization tests. In vitro studies demonstrated that CRP-CDs exhibited excellent biocompatibility. CRP-CDs treatment effectively reduced intracellular TG and TC levels. Administration of CRP-CDs alleviated elevated serum biochemical parameters in HFD-induced MASH mice. Histological examination further revealed the protective effect of CRP-CDs on liver damage induced by HFD feeding. In addition, CRP-CDs decreased hepatic levels of IL-1β, IL-6, TNF-α, and MDA, and enhanced SOD activity as well as GSH content. Analysis of proteomic results demonstrated that CRP-CDs alleviated MASH pathogenesis via synergistic modulation of multiple signaling cascades rather than acting on a single pathway. Moreover, in vivo toxicity assessment validated the favorable systemic biosafety of CRP-CDs with no obvious adverse toxic effects in mice. In conclusion, novel CRP-CDs represent an environmentally friendly and biocompatible nanomaterial capable of effectively alleviating HFD-triggered MASH, which shows promise as a candidate for preclinical therapeutic development against fatty liver disease. Notably, these results are derived from preclinical short-term animal studies, and further validation is required prior to clinical translation.

## 1. Introduction

Metabolic dysfunction-associated steatohepatitis (MASH), formerly termed non-alcoholic steatohepatitis (NASH), is now recognized as the inflammatory and progressive subtype of metabolic dysfunction-associated steatotic liver disease (MASLD). With 1.3 billion individuals affected worldwide in 2023, accounting for 16.1% of the global population, MASLD poses considerable ongoing and prospective health and economic burdens [1]. MASH is characterized by the presence of hepatic steatosis accompanied by lobular inflammation and ballooning degeneration of hepatocytes, with or without fibrosis [2]. As the disease progresses, MASH may advance to cirrhosis and liver failure, which substantially elevates the risk of HCC development [3,4]. With the increasing global prevalence of obesity, MASH represents the most rapidly expanding risk factor for liver cancer [5]. Resmetirom, the first FDA-approved oral drug for MASH, exerted prominent improvements in hepatic lesions and lipid metabolism in a phase III trial of 966 patients [6]. Despite its satisfactory therapeutic performance, the frequent gastrointestinal adverse reactions greatly impair patient treatment tolerance [7]. Therefore, it is critical to explore novel therapeutic agents against MASH with potent efficacy and improved safety characteristics.

Carbon dots (CDs), a class of zero-dimensional carbon nanomaterials with a diameter of <10 nm, possess unique advantages including small particle size, excellent biocompatibility, high water solubility, and facile surface functionalization [8,9]. In recent years, Traditional Chinese medicine (TCM) features abundant sources, mild pharmacological effects, and multi-component and multi-target therapeutic characteristics, which have been widely applied in the prevention and clinical intervention of various chronic diseases. Based on the combination of TCM resources and nanotechnology, TCM-derived carbon dots (TCM-CDs) are successfully fabricated through eco-friendly and facile green synthesis routes, with natural TCM herbs or their bioactive components serving as carbon precursors [10]. Such a green synthesis strategy enables TCM-CDs to perfectly inherit and retain the core pharmacological activities of TCM precursors, while simultaneously integrating the unique nanoscale advantages of carbon dots. Previous studies have demonstrated that TCM-CDs exert excellent regulatory and therapeutic effects on multiple pathological processes, especially metabolic disorders and inflammatory responses [11,12,13,14]. Accordingly, TCM-CDs have emerged as promising novel nanotherapeutic agents for the treatment and intervention of metabolic and inflammatory diseases, providing new insights for the development of high-efficiency and low-toxicity biomedical therapeutic materials.

*Citri Reticulatae Pericarpium* (CRP), as a classic medicinal and edible TCM, has been traditionally applied for the amelioration of liver disorders [15,16,17]. It is rich in flavonoids, polyphenols, and other bioactive carbon-containing components [18]. Nevertheless, the poor aqueous solubility and low oral bioavailability of these citrus flavonoids greatly restrict their clinical translation, which highlights the demand for alternative formulation strategies [19,20,21]. In this context, CRP serves as an ideal low-cost, green, and biocompatible precursor for the fabrication of functional CDs. Here, in this study, CRP can be successfully converted into CRP-derived carbon dots (CRP-CDs). We evaluated the in vitro biosafety of CRP-CDs and investigated their regulatory effect on hepatic lipid accumulation using an FFA-induced fatty-degeneration cell model. Furthermore, we explored the protective actions of CRP-CDs against HFD-induced MASH. Using DIA-MS quantitative proteomics, we further delineated the core regulatory signaling pathways responsible for such protection, alongside a comprehensive evaluation of the in vivo systemic biosafety of CRP-CDs. This study seeks to provide a novel low-toxicity natural nanotherapeutic candidate for MASH treatment.

## 2. Result

### 2.1. Synthesis and Characterization of CRP-CDs

**Figure 1:**
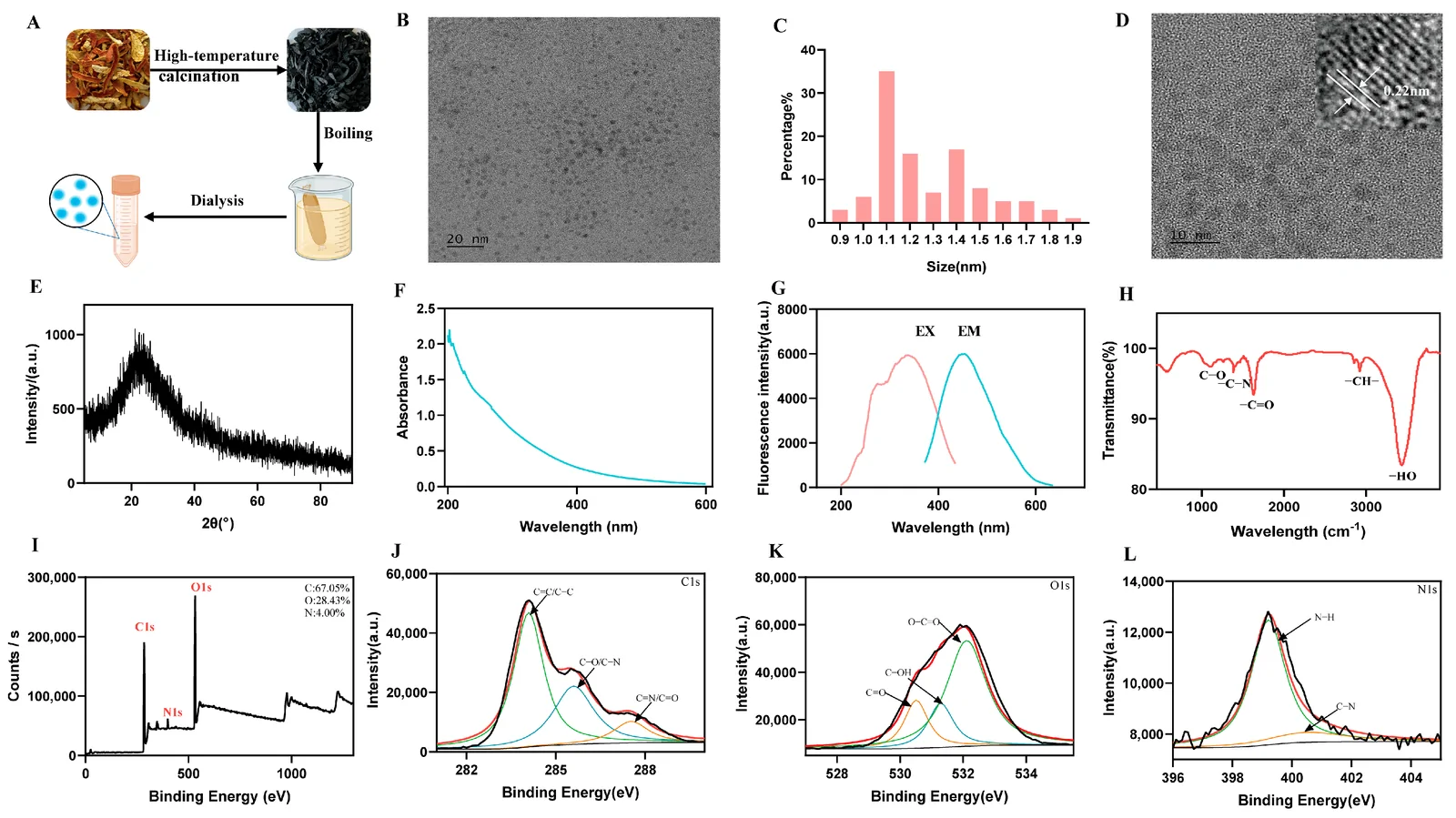
Preparation and characterization of CRP-CDs. (**A**) The preparation process of CRP-CDs. (**B**,**C**) TEM images and particle size distribution histogram of CRP-CDs. (**D**) The high-resolution TEM images of CRP-CDs. (**E**) XRD of CRP-CDs. (**F**) UV-vis spectrum of CRP-CDs. (**G**) Fluorescence performance of CRP-CDs. (**H**) FTIR spectroscopy of CRP-CDs. (**I**) High-resolution XPS spectra of CRP-CDs. (**J**) High-resolution XPS C 1s spectrum of CRP-CDs. (**K**) High-resolution XPS O 1s spectrum of CRP-CDs. (**L**) High-resolution XPS N 1s spectrum of CRP-CDs.

We employed *Citri Reticulatae Pericarpium*, a traditional Chinese medicine with excellent biological activity, as the carbon source to synthesize CRP-CDs through high-temperature calcination. The preparation process of CRP-CDs is shown in Figure 1A. Transmission electron microscopy (TEM) revealed that the as-prepared CRP-CDs exhibited good dispersion and possessed a spherical morphology. The particle diameters ranged from 0.70 to 1.90 nm, with an average diameter of 1.30 ± 0.23 nm, and all sizes were less than 10 nm (Figure 1B,C). The high-resolution TEM image revealed a lattice spacing of 0.22 nm that corresponds to the graphitic (100) plane, which only exists in isolated tiny sp^2^ graphitic nanodomains (Figure 1D). The XRD pattern showed a broad diffraction signal at 21–23°, which was attributed to the (002) plane and confirmed the amorphous structure of carbon dots (Figure 1E). In the full-spectrum scanning range, no obvious characteristic absorption peaks were observed in the ultraviolet and visible regions (Figure 1F). Fluorescence (FL) performance characterization of CRP-CDs demonstrates that under the optimal excitation wavelength of λ_ex_ = 339 nm, the CRP-CDs emit a strong, sharp characteristic fluorescence peak at λ_em_ = 453 nm with a symmetric profile. Notably, the CRP-CDs exhibit a large Stokes shift of 114 nm, which efficiently mitigates spectral overlap between the excitation and emission bands (Figure 1G). Complementarily, FT-IR spectroscopy revealed the presence of diverse functional groups on the CRP-CD surface, including -C=O, -OH, -CN, and C–O (Figure 1H). The XPS further confirmed the chemical composition and surface states of the CRP-CDs. The survey spectrum identified the presence of C, O, N, and P as the main constituent elements (atomic percentages: C—67.05%, O—28.43%, N—4.00%, P—0.52%), while high-resolution XPS spectra of the C1s, O1s, and N1s regions provided detailed insights into the chemical bonding configurations (Figure 1I). Specifically, the C1s spectrum could be deconvoluted into three distinct peaks at 284.09 eV (C=C/C–C), 285.6 eV(C–O/C-N), and 287.53 eV(C-N/C=O). The O1s spectrum showed three peaks at 530.51 eV (C=O), 531.29 eV (C-OH), and 532.11 eV (O=C–O), consistent with the functional groups identified by IR spectroscopy. Additionally, the N1s spectrum exhibited a peak at 399.21 eV (N-H) and 400.5 eV(C-N), attributed to pyrrolic/pyridinic N, which further enriches the surface electronic states of the CRP-CDs (Figure 1J–L). The zeta potential of CRP-CDs was +1.8 mV, indicating nearly neutral surface charge (Figure S1).

### 2.2. Biological Activity Evaluation of CRP-CDs In Vitro

**Figure 2:**
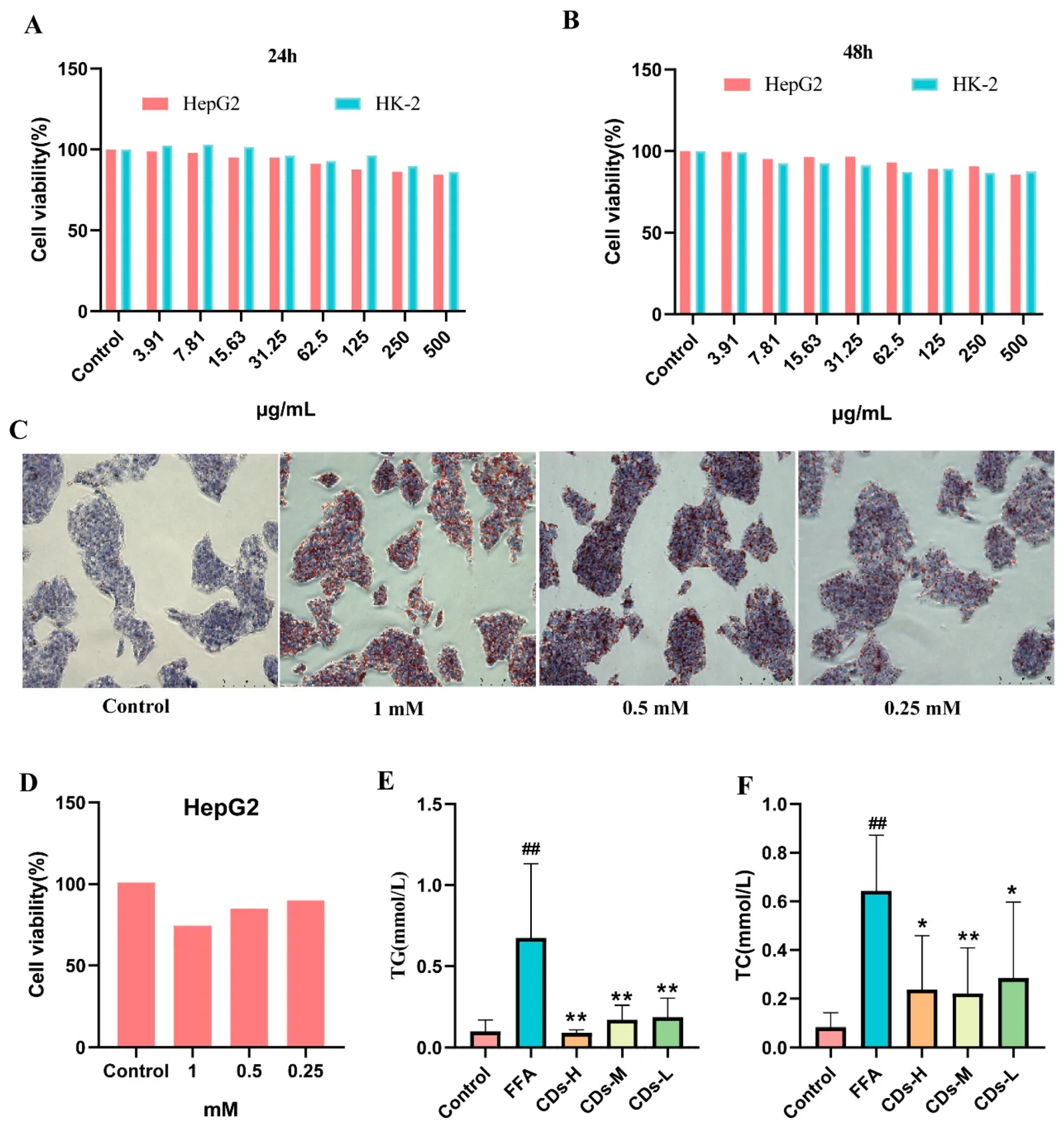
Biological activity evaluation of CRP-CDs. (**A**,**B**) Cell viability of HepG2 and HK-2 cells after incubation with gradient concentrations of CRP-CDs for 24 h/48 h. (**C**) Oil Red O staining of HepG2 cells after treatment with 0.25, 0.5, and 1 mM FFA. (**D**) Cell viability of HepG2 after treatment with 0.25, 0.5, and 1 mM FFA. (**E**) TG levels in HepG2 cells across different experimental groups. (**F**) TC levels in HepG2 cells across different experimental groups. The data represent the mean ± SD; ^##^ *p* < 0.01 as compared to the normal control group. * *p* < 0.05 and ** *p* < 0.01 as compared to the model group (model: FFA-induced untreated group; CDs-H: high; M: medium; L: low). H, M, and L = treatment with CRP-CDs at 125, 62.5, and 31.25 μg/mL, respectively.

The in vitro biological activity of CRP-CDs was evaluated using HepG2 and HK-2 cells. The CCK-8 assay was performed to evaluate the cytotoxicity of CRP-CDs at a series of gradient concentrations (3.91, 7.81, 15.63, 31.25, 62.5, 125, 250, and 500 μg/mL). After treatment with CRP-CDs at concentrations up to 500 μg/mL for 24 and 48 h, cell viability remained above 85%, suggesting the prominent in vitro biocompatibility of CRP-CDs (Figure 2A,B). To further evaluate the protective effect of CDs against cellular lipid accumulation, an FFA-induced hepatocyte steatosis model was established in HepG2 cells. Oil Red O staining and CCK-8 assays showed that 0.5 mM FFA markedly induced intracellular lipid accumulation, while cell viability remained above 80% with no apparent cytotoxicity. Accordingly, 0.5 mM FFA was determined as the optimal concentration for establishing the intracellular lipid accumulation model (Figure 2C,D). As shown in the results, FFA stimulation markedly induced lipid accumulation in HepG2 cells. Compared with the control group, the FFA model group showed significantly increased TG and TC levels (*p* < 0.01). After treatment with CRP-CDs (H, M, and L doses = 125, 62.5, and 31.25 μg/mL), intracellular TG and TC contents were prominently decreased (*p* < 0.01) (Figure 2E,F). These results demonstrate that CRP-CDs possess excellent in vitro biocompatibility and can effectively ameliorate FFA-induced abnormal lipid metabolism. This work provides preliminary experimental evidence for their potential application in metabolic disorders such as MASH.

### 2.3. CRP-CDs Attenuate HFD-Induced MASH in Mice

**Figure 3:**
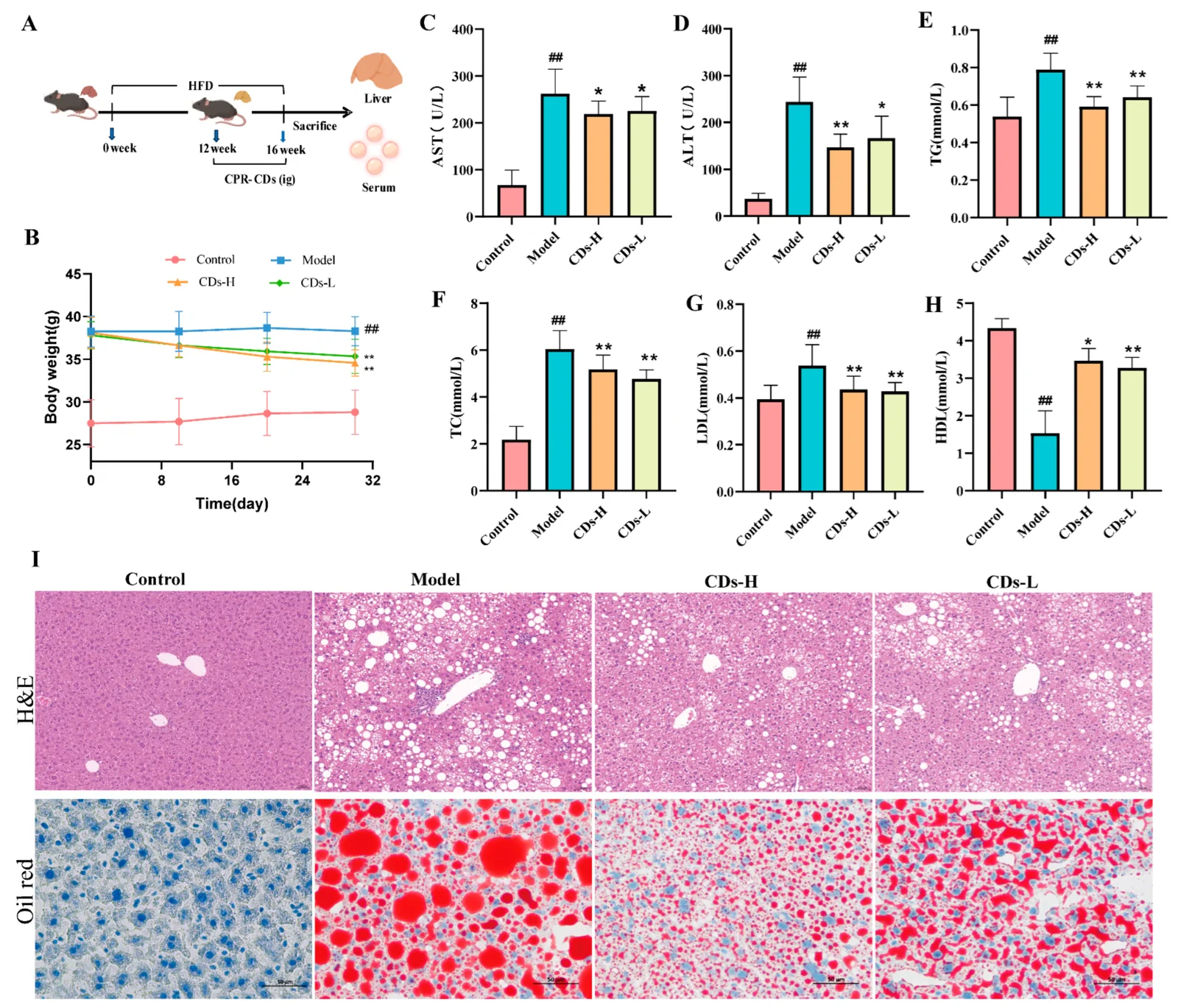
Protective effects of CRP-CDs on HFD-induced MASH. (**A**) Schematic diagram of HFD-induced MASH mouse model establishment and CRP-CD intervention. C57BL/6 mice were fed with HFD for 16 weeks to construct the MASH model. From the 12th week to the 16th week, mice were administered CRP-CDs by gavage for 4 consecutive weeks. All mice were sacrificed at week 16 for subsequent experimental detection. (**B**) Body weight changes in mice after administration. (**C**,**D**) Serum AST and ALT levels in mice. (**E**,**F**) Serum TG and TC levels in mice. (**G**,**H**) Serum LDL and HDL levels in mice. (**I**) Representative images of H & E staining (200×) and Oil Red O staining (400×) of liver sections. The data represent the mean ± SD; ^##^ *p* < 0.01 as compared to the normal control group (*n* = 6). * *p* < 0.05 and ** *p* < 0.01 as compared to the model group (model: HFD-induced MASH untreated group, *n* = 8; H: high; L: low). H and L doses = treatment with CRP-CDs at 5.00 and 1.25 mg/kg, *n* = 9 each, respectively.

To explore the regulatory effect of CRP-CDs on HFD-induced MASH in vivo, mice were fed a high-fat diet for 16 consecutive weeks to establish a MASH model. Starting at week 12 and lasting until week 16 of modeling, mice were given daily intragastric administration of CRP-CDs for 4 consecutive weeks (Figure 3A). The MASH model group exhibited significantly higher body weight compared with the normal control group, while CRP-CDs intervention markedly reduced the elevated body weight (*p* < 0.01) (Figure 3B). Compared with the control group, the model group exhibited significantly increased serum levels of AST, ALT, TG, TC, and LDL, and decreased HDL levels (## *p* < 0.01). Notably, compared with the model group, the CRP-CDs treatment group presented obvious reductions in AST, ALT, TG, TC, and LDL levels (* *p* < 0.05), as well as an obvious elevation in HDL level (* *p* < 0.05), indicating that the intervention could improve liver function and lipid metabolism disorder (Figure 3C–H).

H & E staining showed that the control group presented intact hepatic cords that were regularly arranged, and hepatocytes displayed normal morphology without obvious cytoplasmic vacuoles. In the model group, hepatic lobule structure was destroyed, with severe hepatocyte steatosis, obvious ballooning degeneration, massive inflammatory cell infiltration, and cell necrosis. CRP-CD administration effectively attenuated hepatic histological lesions, suppressed hepatocyte vacuolization and ballooning degeneration, and mitigated inflammatory cell recruitment and infiltration. Oil Red O (ORO) staining was performed to investigate the effects of CRP-CDs on hepatic steatosis in MASH mice. A large number of red lipid droplets accumulated in the liver of HFD-fed mice, accompanied by severe hepatic lipid deposition. The ORO-positive staining area was significantly higher than that in the control group. Quantitative analysis of Oil Red O staining revealed that the lipid droplet area fraction was significantly increased in the model group compared with the control group. Treatment with CRP-CDs markedly reduced the lipid droplet area fraction, indicating alleviation of hepatic lipid accumulation (Figure S2). Notably, the CRP-CDs treatment group had an obviously reduced number and area of lipid droplets, inhibited abnormal hepatic lipid accumulation, and improved hepatic steatosis (Figure 3I). Overall, CRP-CDs effectively improved lipid profiles and liver function in MASH mice.

### 2.4. CRP-CDs Inhibit Liver Inflammation and Oxidative Stress

**Figure 4:**
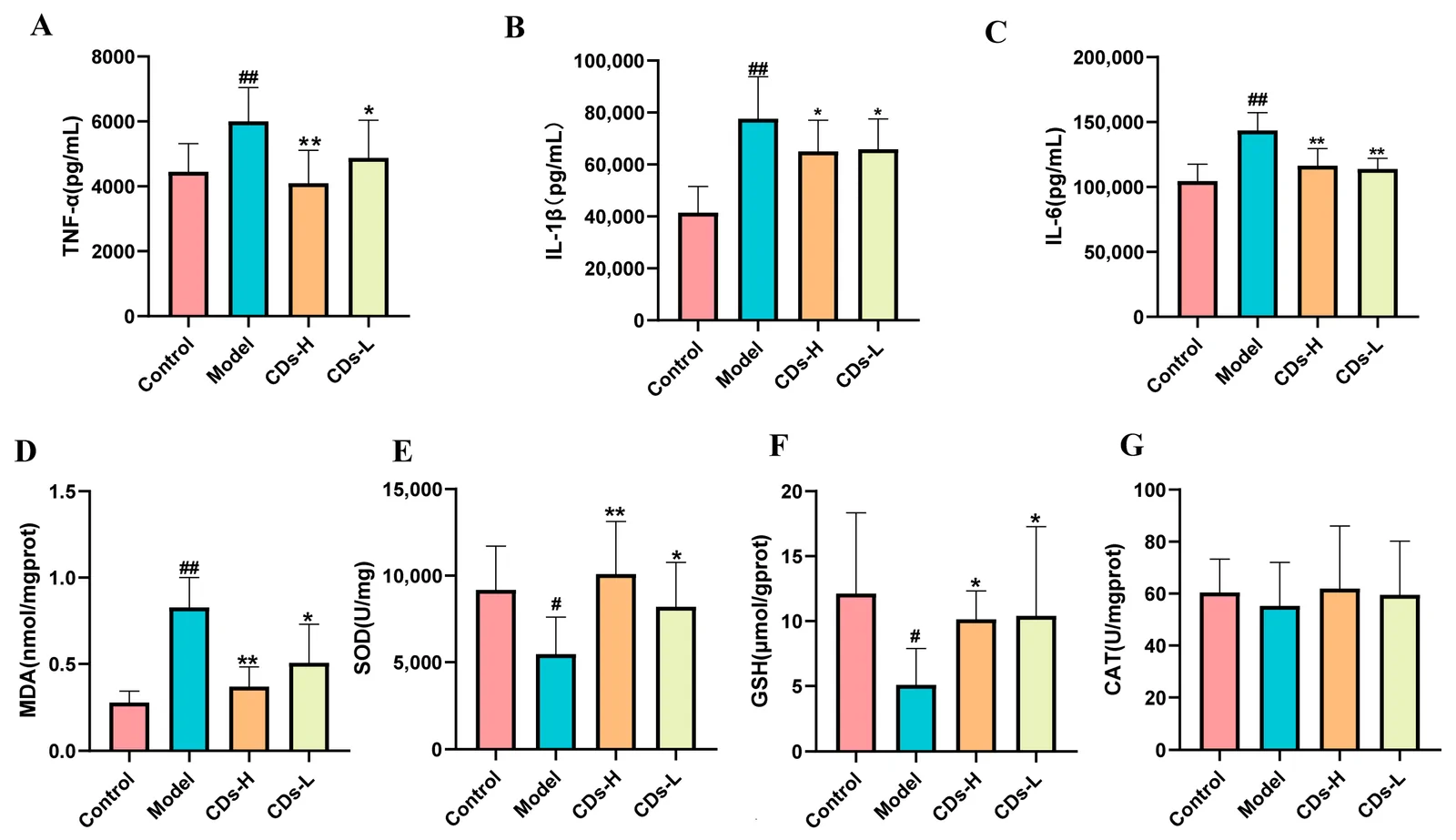
Effects of CRP-CDs on hepatic inflammation and oxidative stress. (**A**) Liver levels of TNF-α quantified by ELISA. (**B**) Liver levels of IL-1β quantified by ELISA. (**C**) Liver levels of IL-6 quantified by ELISA. (**D**) Liver MDA level. (**E**) Liver SOD level. (**F**) Liver GSH level. (**G**) Liver CAT level. The data represent the mean ± SD; ^#^ *p* < 0.05 and ^##^ *p* < 0.01 as compared to the normal control group. * *p* < 0.05 and ** *p* < 0.01 as compared to the model group (model: HFD-induced MASH untreated group; H: high; L: low). H and L doses = treatment with CRP-CDs at 5.00 and 1.25 mg/kg, respectively.

Long-term HFD feeding successfully induced MASH pathological characteristics in mice. HFD challenge markedly triggered a persistent hepatic inflammatory response and excessive oxidative stress in liver tissues. Meanwhile, abnormal lipid accumulation further aggravated hepatocyte injury, inflammatory cell infiltration, and oxidative stress imbalance, ultimately accelerating the progression of hepatic steatosis to MASH. Compared with the control group, the HFD-induced MASH model group exhibited significantly elevated levels of pro-inflammatory cytokines, including TNF-α, IL-1β, and IL-6, as well as the lipid peroxidation product MDA in liver tissue (^#^ *p* < 0.05, ^##^ *p* < 0.01) (Figure 4A–D). Meanwhile, the activities of SOD and GSH levels were significantly decreased (*p* < 0.05, *p* < 0.01) (Figure 4E,F). However, there was no significant difference in CAT activity between the model group and the control group (*p* > 0.05) (Figure 4G). In contrast, after intervention with CRP-CDs, the treatment group showed a significant reduction in TNF-α, IL-6, IL-1β, and MDA levels, while SOD activity and GSH content were significantly higher in the treatment group versus the model group (*p* < 0.05). Notably, CAT activity exhibited no statistically significant variation among the control, HFD-induced MASH model, and treatment groups (*p* > 0.05).

### 2.5. Proteomics Analysis in Liver Tissue

**Figure 5:**
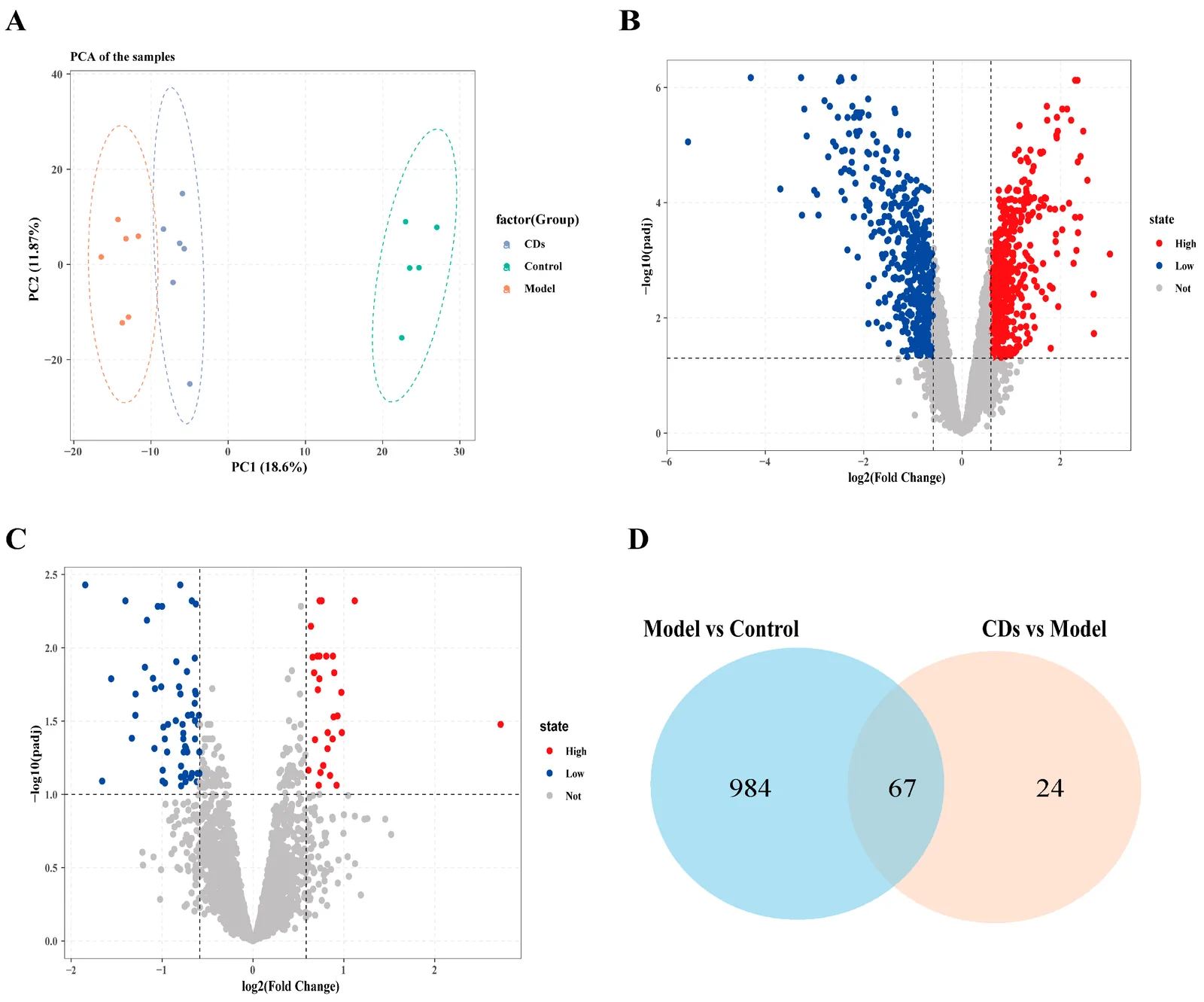
Proteomic analysis of CRP-CDs intervention in HFD-induced MASH. (**A**) PCA of liver samples from control, model, and CRP-CDs groups. (**B**) Volcano plot of protein statistics of liver samples from model and control groups. (**C**) Volcano plot of protein statistics of liver samples from CRP-CDs and model groups. (**D**) Differential gene Venn diagram of liver samples from control, model, and CRP-CDs groups.

**Figure 6:**
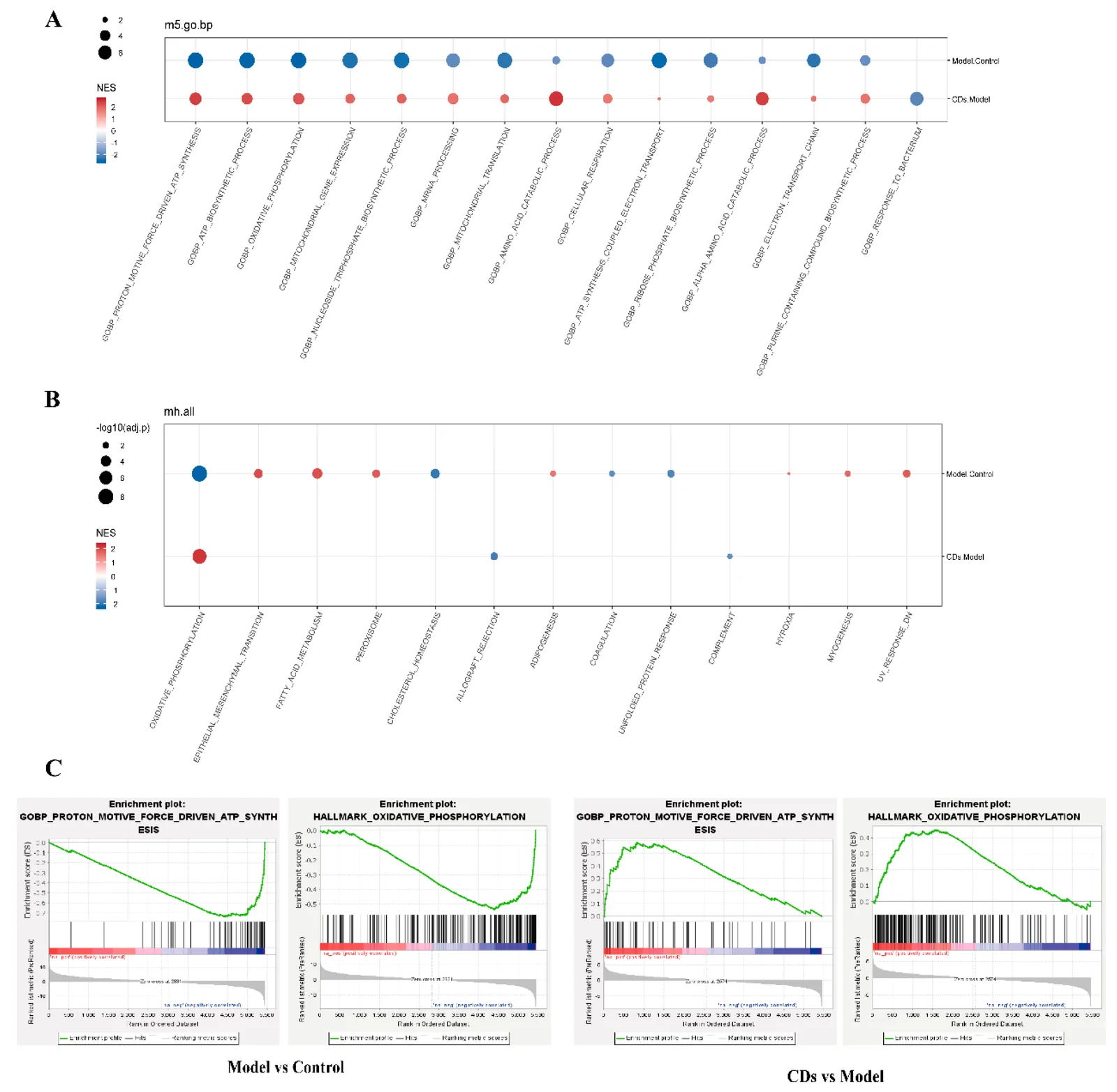
Gene set enrichment analysis. (**A**) GSEA enrichment curve of the top altered GO term from MSigDB C5 (M5) gene sets for hepatic proteome in CRP-CDs-treated HFD-MASH mice. (**B**) GSEA bubble plot of MSigDB MH (Mouse Hallmark) gene sets for the hepatic proteome in CRP-CDs-treated HFD-MASH mice. NES, normalized enrichment score. (**C**) Gene set enrichment analysis plot highlighting activation of ATP synthesis and oxidative phosphorylation pathways.

In order to explore the pharmacological mechanism by which CRP-CDs exert therapeutic effects on MASH, we adopted DIA-MS to perform in-depth proteomic profiling of liver tissues harvested from mice assigned to the control, model, and CRP-CD treatment groups. A total of 23 samples were initially subjected to proteomic analysis. After quality control, 17 samples were retained for the remaining analyses. Figure 5A presents the overall proteomic landscape of liver tissues from the control, model, and CRP-CD groups. Principal component analysis (PCA) demonstrated distinct separation among the three experimental groups, indicating significant overall proteome differences induced by pathological modeling and CRP-CDs intervention. To further characterize differentially expressed proteins (DEPs), volcano plots were generated for pairwise comparisons, including model vs. control and CRP-CDs vs. model, which visually displayed the distribution of upregulated and downregulated proteins in each comparison (Figure 5B,C). The results analysis revealed that a total of 1051 DEPs were screened out in the model group relative to the control group (Table S1), while 91 DEPs were identified in the CRP-CDs-treated group compared with the model group (Table S2). Additionally, Venn diagram analysis was performed to analyze the overlap of DEPs from the two pairwise comparison groups, and the results showed that 67 common DEPs were shared between the two comparison sets (Table S3), intuitively reflecting the core protein changes responding to disease modeling and CRP-CDs intervention (Figure 5D). GSEA was performed on two distinct gene set collections from MSigDB, namely the C5 (M5) Gene Ontology gene sets and MH (Mouse Hallmark) gene sets. Enrichment curves were plotted for the most significantly perturbed GO biological processes. GSEA results showed global inhibition of mitochondrial energy metabolism pathways (oxidative phosphorylation, electron transport chain, ATP synthesis, mitochondrial transcription and translation) in MASH liver tissue, accompanied by disturbed nucleotide/amino acid metabolism and hyperactivated bacterium-responsive immune pathways. These transcriptional disturbances cooperatively induce mitochondrial bioenergetic failure and severe hepatic inflammation. CRP-CDs intervention normalized the above dysregulated pathways to mitigate MASH pathological progression (Figure 6A). Notably, bubble plots visualizing GSEA outcomes of hallmark gene sets indicated that oxidative phosphorylation was among the most prominently enriched pathways, implying potential regulatory effects of CRP-CDs on this biological process (Figure 6B). Collectively, these observations suggest that mitochondrial energy homeostasis is markedly disrupted in the disease model, and CRP-CDs intervention may help restore such pathological disturbance (Figure 6C).

### 2.6. Biosafety Evaluation of CRP-CDs In Vivo

**Figure 7:**
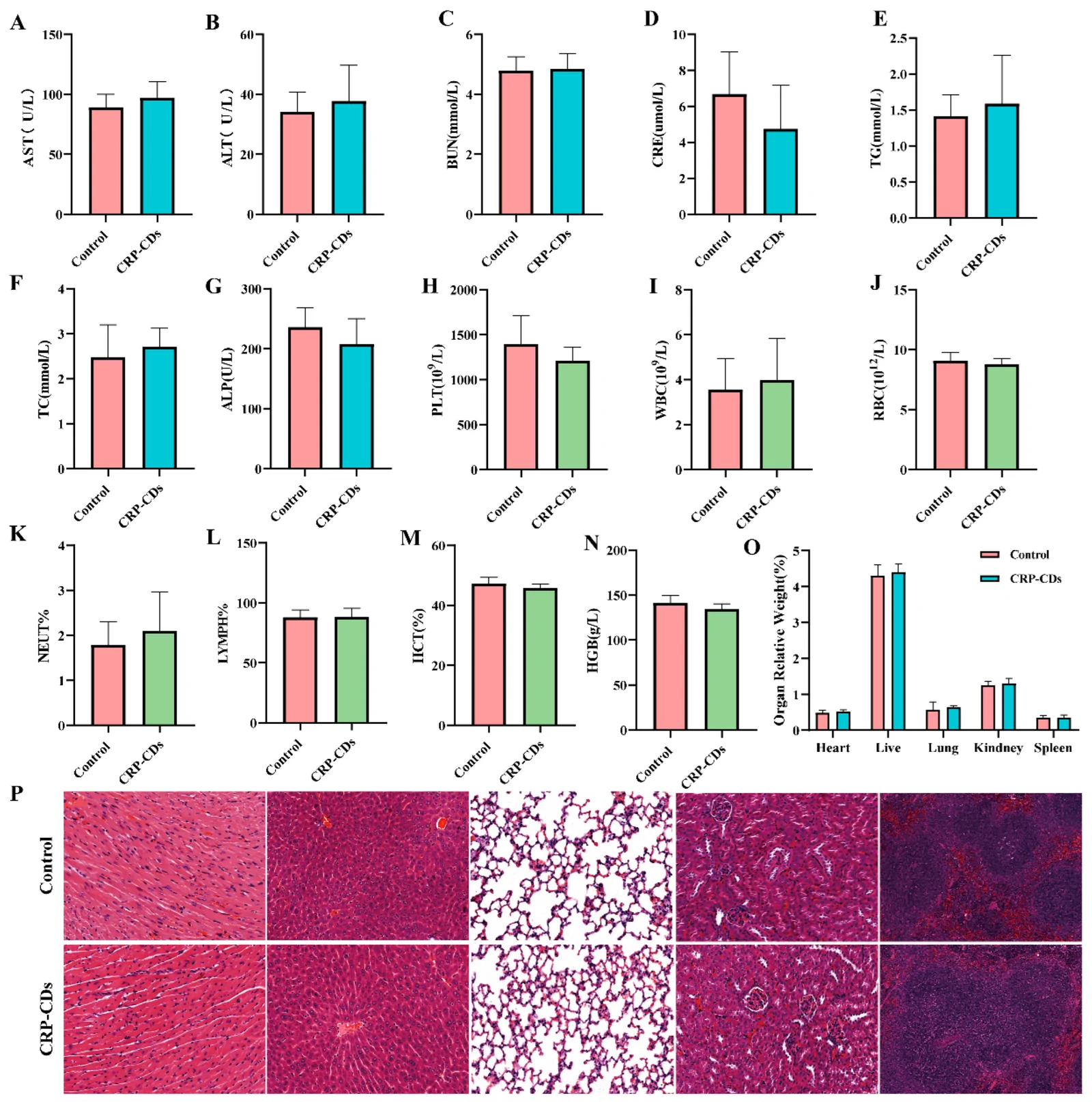
Biosafety evaluation of CRP-CDs. (**A**–**G**) Serum ALT, AST, BUN, CRE, TG, TC, and ALP levels. (**H**–**N**) Routine blood PLT, WBC, RBC, NEUT%, LYMPH%, HCT, and HGB levels. (**O**) Visceral index levels in control and CRP-CDs groups (heart, liver, lung, kidneys, and spleen). (**P**) Histological evaluation of tissue samples (heart, liver, lung, kidneys, and spleen) after administration of CRP-CDs, stained with H&E. White blood cell count (WBC), red blood cell count (RBC), hemoglobin (HGB), hematocrit (HCT), platelet count (PLT), neutrophil percentage (NEUT%), and lymphocyte percentage (LYMPH%).

In vivo safety evaluation of CRP-CDs was performed via intravenous injection of a high concentration (20.85 mg/kg) into mice for one week. Serum biochemical analysis showed no significant abnormalities in liver function indices (ALT and AST), kidney function indices (BUN and CRE), or other key biochemical parameters (TG, TC, and ALP) compared with the control group (Figure 7A–G). Additionally, routine blood tests revealed normal levels of white blood cells, red blood cells, platelets, hemoglobin, and other hematological indices, indicating no adverse effects on the hematopoietic system (Figure 7H–N). Organ coefficient analysis of major organs (heart, liver, lung, kidney, and spleen) showed no statistically significant differences between the CRP-CDs-treated group and the control group (Figure 7O). Moreover, H & E staining of these organs revealed intact tissue structures, no obvious inflammatory infiltration, cell necrosis, or pathological lesions (Figure 7P). These results comprehensively confirm the excellent biocompatibility of CRP-CDs, providing critical support for their potential applications in biomedicine.

## 3. Discussion

As the global prevalence of MASH increases, it has become a critical threat to metabolic health. However, current pharmacological interventions are often limited by substantial side effects and an inability to provide safe, long-term management [22]. Traditional Chinese medicine-derived carbon dots (TCM-CDs) are prepared via green synthetic approaches using natural herbs as carbon precursors. Featuring low toxicity and excellent biocompatibility, these nanomaterials retain the inherent bioactivities of original herbal materials, making them highly promising novel therapeutic agents [23,24]. In the present study, green-synthesized CRP-CDs were successfully prepared from CRP as a natural carbon precursor via carbonization. The CRP-CDs exhibited a relatively uniform size distribution of quasi-spherical particles with diameters under 10 nm and good dispersion. Structural characterizations confirm that CRP-CDs are richly decorated with various oxygen-containing functional groups on their surface, which can enhance their antioxidant capacity and biocompatibility [25]. Optical spectra further confirmed the typical fluorescence properties of carbon dots [24]. In addition, biocompatibility constitutes the primary prerequisite for the biomedical translation of nanomaterials. In vitro cytotoxicity assays based on HepG2 and HK-2 cells verified that CRP-CDs exhibit negligible cytotoxicity within the effective therapeutic concentration range, indicating good cellular compatibility. Meanwhile, in vivo biocompatibility evaluation further indicated that CRP-CDs do not cause obvious damage to major organs and physiological functions in mice. No obvious abnormalities in blood routine and biochemical markers were observed after acute administration, suggesting that CRP-CDs did not induce severe subclinical hematopoietic, hepatic, or renal injury under the tested conditions. Owing to their favorable low-toxicity characteristics, CRP-CDs display promising merits for chronic disease intervention. Nevertheless, long-term repeated-dose toxicity studies are still required to fully confirm their biosafety profile. In this study, a methodological discrepancy in administration routes exists between the MASH therapeutic model and acute toxicity assessment, and we acknowledge this mismatch as a limitation of the present study. The intraperitoneal (i.p.) acute toxicity assay served as an initial screening test to assess the systemic toxicity potential of CRP-CDs following systemic absorption and cannot replace repeated-dose oral toxicity evaluation matching the long-term therapeutic regimen. As previously documented, i.p. administration enables rapid absorption through peritoneal capillaries and lymphatics and is widely adopted for preliminary systemic toxicity screening in rodents [26]. In vivo imaging studies of nanomaterials further demonstrate that i.p.-injected nanoparticles access the bloodstream and major organs (liver, kidney, and spleen) within 0.5–3 h [27]. Therefore, our current i.p. toxicity data cannot fully reflect the in vivo safety profile of orally administered CRP-CDs.

As a widely utilized herb in clinical traditional Chinese medicine, CRP possesses the capacity to ameliorate and regulate pathological liver injuries effectively [15]. Studies have reported that excessive hepatic lipid accumulation is the initiating and core pathological feature of MASH, characterized by abnormally elevated TC and TG. This lipid overload further triggers inflammatory cell infiltration and liver tissue injury [28,29]. In this study, an FFA-induced HepG2 fatty-degeneration cell model was used to simulate the in vitro pathological state of hepatic steatosis. The results demonstrated that CRP-CDs could effectively reduce TC and TG, indicating their ability to reverse lipid metabolic disorder and mitigate hepatic steatosis. These findings provide preliminary experimental support for the potential application of CRP-CDs in metabolic disorders including MASH, prompting us to further investigate their protective effects against MASH progression in vivo. However, this approach has inherent limitations associated with its in vitro cell experiments, which were conducted using HepG2 cells. HepG2 is a tumor-derived human hepatoblastoma cell line with aberrant basal lipid metabolism and suppressed cytochrome P450 expression, features that may alter cellular responses to lipid stress and CRP-CDs intervention. Nevertheless, due to its human genetic background and stable lipid-accumulating capacity, HepG2 cells remain a widely used cell model for preliminary mechanistic investigations of MASH [30].

The HFD-induced MASH mouse model represents a classic preclinical model that recapitulates the core pathological features of human MASH [31,32]. Long-term continuous HFD administration leads to massive lipid droplet accumulation in hepatocytes, accompanied by obvious lobular inflammatory infiltration and hepatocyte ballooning. Histopathological observation revealed prominent hepatic steatosis, excessive hepatic lipid deposition, and inflammatory cell infiltration in liver tissues of HFD-induced MASH mice. Consistently, serum biochemical assays demonstrated elevated levels of liver injury-related markers, reflecting the establishment of hepatic damage in this MASH model. After CRP-CDs intervention, these pathological alterations were markedly alleviated, alongside reduced serum biochemical indicators of hepatic injury, supporting the hepatoprotective effects of CRP-CDs against MASH. Moreover, the liver displayed markedly increased levels of canonical pro-inflammatory cytokines TNF-α, IL-6, and IL-1β, together with elevated MDA content, a critical end-product of lipid peroxidation. The activities of major antioxidant enzymes SOD and the level of GSH were markedly suppressed in the model group. These drastic alterations in inflammatory and oxidative stress biomarkers strongly indicated that HFD intervention effectively induced robust hepatic inflammatory activation and impaired endogenous antioxidant defense systems, which simulated the inflammatory and oxidative pathological phenotypes of MASH [33,34,35]. The present study confirms that CRP-CDs exert potent protective effects against HFD-triggered hepatic steatosis and inflammatory lesions. These nanoparticles markedly lowered serum biochemical indicators, decreased hepatic pro-inflammatory cytokines (IL-1β, IL-6, and TNF-α) as well as the lipid peroxidation product MDA, and simultaneously upregulated SOD activity and GSH content to reconstruct hepatic antioxidant defense systems. These findings verified that CRP-CDs ameliorate MASH-related lesions through multi-dimensional effects, including improving lipid deposition, inhibiting inflammation, and relieving oxidative stress, which may be attributed to the abundant functional groups on the surface of the nanoparticles.

To further dissect the molecular mechanism underlying the MASH-protective effects of CRP-CDs, DIA-MS quantitative proteomics was performed to systematically screen differentially expressed proteins in MASH liver tissues following CRP-CDs intervention. Integrated proteomic analysis identified 67 overlapping differentially expressed proteins altered in MASH mice and reversed by intervention. These overlapping proteins exhibited reciprocal expression changes: proteins upregulated in the HFD-induced MASH model were largely downregulated after treatment, whereas proteins suppressed in the MASH state were restored toward the levels observed in control animals. Consistent with this, given the relatively small pool of differentially expressed proteins, we conducted GSEA using the full quantified proteome. Gene set enrichment analysis identified notable enrichment within mitochondrial energy metabolism pathways. Our results demonstrated that CRP-CDs intervention markedly alleviates lipid deposition, inflammatory infiltration, and oxidative damage in cellular and animal models. Under pathological MASH conditions, massive lipid buildup within hepatocytes severely disrupts mitochondrial respiration. Impaired oxidative phosphorylation collapses transmembrane proton motive force and blunts ATP biosynthesis, leading to widespread mitochondrial bioenergetic failure. Such energy deficiency subsequently amplifies hepatic oxidative stress and robust pro-inflammatory cascades, forming a vicious cycle that accelerates MASH deterioration [36]. Nevertheless, these proteomic observations only demonstrate correlative associations and cannot establish causality; altered protein abundance may reflect secondary adaptive responses rather than the primary driver of liver protection. This study has limitations. Our proteomic and GSEA results only support correlative associations between CRP-CDs-mediated protection and perturbed mitochondrial energy metabolism, without orthogonal validation of key proteins or mitochondrial functional assays. Hence, causality cannot be established, and it remains uncertain whether these proteomic shifts are direct molecular responses or secondary adaptive events. Further work is required to validate candidate proteins and explore deeper mitochondrial functional alterations to fully delineate the precise mechanism underlying CRP-CDs’ action against MASH.

## 4. Materials and Methods

### 4.1. Materials

*Citri Reticulatae Pericarpium* (CRP) was obtained from Beijing Qiancao Herbal Pieces Co., Ltd. (Beijing, China). Dialysis membranes (molecular weight cut-off was 1000 Da) were purchased from Beijing Ruida Henghui Technology Development Co., Ltd. (Beijing, China). The cell counting kit-8 (CCK-8) was brought from Dojindo Molecular Technologies, Inc. (Kumamoto, Japan). Dulbecco’s Modified Eagle Medium (DMEM) and fetal bovine serum (FBS) were purchased from Mediatech, Inc. (Corning, NY, USA). Absolute ethanol (EtOH) solution was purchased from Sigma-Aldrich Co. (St. Louis, MO, USA). Lowry protein assay kit was purchased from Solarbio Science & Technology co., LTD (Beijing, China). Superoxide dismutase (SOD), catalase (CAT), glutathione (GSH), malondialdehyde (MDA), total cholesterol (TC), and triglyceride (TG) kits were purchased from Jiancheng Bioengineering Institute (Nanjing, China). ELISA kits to measure tumor necrosis factor-α (TNF-α), interleukin 6 (IL-6), and interleukin 1β (IL-1β) were purchased from Elabscience (Wuhan, China). Deionized water was used as a solvent for all experiments.

### 4.2. Preparation of Carbon Dots

Carbon dots (CDs) were prepared from CRP via a modified carbonization method combined with aqueous extraction and purification. First, 80.0 g of dried CRP raw material was accurately weighed and loaded into a corundum crucible. The crucible was placed in a muffle furnace (TL0612, Beijing Zhong Ke Aobo Technology Co., Ltd., Beijing, China) and heated to 350 °C at a heating rate of 5 °C/min. The carbonization process was maintained at 350 °C for 1 h to obtain CRP carbonisata. After the carbonization process, the muffle furnace was turned off, and the crucible was allowed to cool naturally to 30 °C (room temperature) to avoid thermal shock-induced structural damage. The CRP carbonisata was then transferred to an agate mortar and ground into a fine powder for subsequent extraction. The ground CRP carbonisata powder was added to distilled water and heated to 100 °C. The mixture was boiled for 1 h, and the extraction process was repeated twice. Second, after extraction, the mixture was cooled to room temperature naturally and filtered through a 0.22 μm filter membrane to remove insoluble large particles and uncarbonized residues. The filtrate was then transferred to a 1000 Da dialysis bag, and dialysis was performed against deionized water for 7 days to remove small-molecule impurities. During dialysis, deionized water was refreshed every 12 h to maintain a stable concentration gradient. Finally, the solution retained inside the dialysis membrane was collected for further characterization. All biological experiments in this study adopted the freshly prepared CRP-CDs solution.

### 4.3. Characterization and identification of CRP-CDs

The morphology, size, and dispersibility of the prepared CRP-CDs were characterized using a JEM-1230 high-resolution TEM (HRTEM, Japan Electron Optics Laboratory, Tokyo, Japan) operated at an acceleration voltage of 200 kV. Prior to testing, the CRP-CD sample was diluted to an appropriate concentration (0.5mg/mL) with ultrapure water and ultrasonicated for 15 min to ensure uniform dispersion. A 1 μL aliquot of the diluted suspension was dropped onto a copper grid coated with a carbon-supported film and then dried naturally at room temperature in a dust-free environment to remove excess solvent. HRTEM images were captured to observe the lattice spacing of CRP-CDs, and the particle size distribution was statistically analyzed using ImageJ software (version 1.8.0) based on more than 100 randomly selected particles.

The crystal structure of CRP-CDs was analyzed by X-ray diffraction (XRD) spectroscopy (Bruker AXS, Karlsruhe, Germany) with Cu Kα radiation (λ = 1.5418Å). The CRP-CDs sample was ground into a fine powder and pressed into a uniform thin sheet on a glass sample holder to ensure flatness and preferred orientation. The XRD patterns were recorded in the 2θ range of 5–90 ° with a step size of 0.02° at 40 kV and 150 mA. The phase composition and crystallinity of CRP-CDs were determined by analyzing the position, intensity, and width of diffraction peaks.

The optical absorption properties of CRP-CDs were measured using an ultraviolet–visible spectrophotometer (UV-Vis, CECIL, Cambridge, UK) in the wavelength range of 200–600 nm. The CRP-CDs sample was diluted with ultrapure water and transferred into a quartz cuvette (1 cm path length) after ultrasonic dispersion for 10 min. Ultrapure water was used as the blank control to eliminate background absorption. The absorption spectrum was recorded at room temperature with a scanning speed of 200 nm/min and a slit width of 2 nm.

Fluorescence properties of CRP-CDs, including emission spectra and excitation spectra, were characterized using an F-4500 fluorescence spectrophotometer (FL, Tokyo, Japan). For emission and excitation spectra measurements, the CRP-CDs sample was placed in a quartz cuvette, and the excitation wavelength was set in the range of 280–400 nm (step size 5 nm), while the emission wavelength was scanned from 300 to 700 nm. The slit widths for excitation and emission were both set to 5 nm, and the scanning speed was 1000 nm/min.

Fourier transform infrared (FTIR) spectroscopy (Thermo Fisher Scientific, San Diego, CA, USA) was employed to characterize the functional groups present on the surface. The CRP-CD sample was mixed with potassium bromide (KBr) at a mass ratio of 1:100, ground into a homogeneous powder, and pressed into a transparent pellet. The FTIR spectrum was recorded in the wavenumber range of 4000–500 cm^−1^.

The elemental composition, chemical valence states, and surface functional groups of CRP-CDs were investigated using an ESCALAB 250Xi X-ray photoelectron spectrometer (XPS, Thermo Fisher Scientific, SanDiego, CA, USA) with Al Kα radiation (hν = 1486.6 eV) as the excitation source. The CRP-CD sample was evenly spread on a clean silicon wafer and dried under vacuum at 60 °C for 2 h to eliminate moisture and adsorbed impurities. The survey scan was performed in the binding energy range of 0–1300 eV with a pass energy of 100 eV and a step size of 1 eV to identify the presence of elements (C, O, and N). High-resolution XPS spectra of C 1s, O 1s, and other relevant elements were collected with a pass energy of 30 eV and a step size of 0.05 eV. The spectra were calibrated using the C 1s peak at 284.8 eV as a reference, and peak fitting was performed using XPS Peak 4.1 software to analyze the chemical bonding configurations.

### 4.4. Functional Evaluation of CRP-CDs In Vitro

The in vitro cytotoxicity of CRP-CDs against human hepatocellular carcinoma cells (HepG2) and human kidney proximal tubular epithelial cells (HK-2) was evaluated using the Cell Counting Kit-8 (CCK-8) assay. HK-2 cells were used to evaluate the renal cytotoxicity of CRP-CDs. Because CRP-CDs are mainly eliminated through renal excretion, HK-2 cells represent a common in vitro model for assessing the renal biocompatibility of nanoparticles. HepG2 and HK-2 were purchased from the Cell Bank of the Chinese Academy of Sciences (Shanghai, China). Cells were cultured in Dulbecco’s Modified Eagle’s Medium (DMEM, Gibco, Grand Island, NY, USA) supplemented with 10% fetal bovine serum (FBS, Gibco, Grand Island, NY, USA) at 37 °C in a humidified incubator with 5% CO_2_. The culture medium was refreshed every 24 h, and cells in the logarithmic growth phase were used for subsequent experiments. Cells were seeded at a density of 8 × 10^3^ cells/well in 96-well plates. CRP-CDs solution after dialysis was freeze-dried to obtain dry powder, which was then dissolved in sterile DMEM to prepare a stock solution at a concentration of 1 mg/mL. The stock solution was sterilized by filtration through a 0.22 μm sterile filter membrane, then serially diluted with complete culture medium to final working concentrations of 3.91, 7.81, 15.63, 31.25, 62.5, 125, 250, and 500 μg/mL. Subsequently, the culture medium was discarded, and the cells were incubated in fresh culture medium containing CRP-CDs at different doses for another 24 h and 48 h. After incubation, the medium containing CRP-CDs was aspirated and discarded. CCK-8 working solution was prepared using fresh culture medium and added to each well (*n* = 6), and the plates were incubated at 37 °C for another 2 h in the dark. The absorbance value (OD) of each well was measured at a wavelength of 450 nm using a microplate reader.

To evaluate the regulatory effect of CRP-CDs on cellular lipid accumulation, in vitro experiments were performed. First, free fatty acid (FFA; oleic acid/palmitic acid mixture, 2:1) concentration screening was conducted using CCK-8 and Oil Red O assays. HepG2 cells were seeded at 8 × 10^3^ cells/well in 96-well plates (for CCK-8) or 1 × 10^4^ cells/well in 12-well plates (for ORO staining) and cultured until adherence. Then, HepG2 cells were incubated with FFA at gradient concentrations (0.25, 0.5, and 1 mM) for 24 h (control, *n* = 4; FFA and treatment groups, *n* = 5). Cell viability was evaluated using the CCK-8 assay, and intracellular lipid accumulation was assessed by Oil Red O (ORO, G1262, Solarbio,Beijing, China) staining following the manufacturer’s protocols. Subsequently, FFA-induced lipid-accumulated HepG2 cells and then CRP-CDs (125, 62.5, and 31.25 μg/mL) were added, followed by 24 h incubation. Cells were washed twice with PBS, then harvested for the detection of triglyceride (TG) and total cholesterol (TC) levels following the manufacturers’ instructions for the respective assay kits.

### 4.5. Establishment of MASH Mouse Model and CRP-CDs Administration

Male mice (C57BL/6, weighting 22 ± 1.0, eight old weeks) were purchased from the Laboratory Animal Center, Si Beifu (Beijing, China), and were provided with a Laboratory Animal Certificate of Conformity. The mice were housed in an environmentally controlled breeding room under conditions of 24.0 ± 1.0 °C, relative humidity of 55–65%, and a 12 h light/12 h dark cycle. All animals were acclimatized for 7 days prior to the experiment with free access to standard rodent chow and water. The groups were divided into a normal control group (*n* = 6), MASH model group (*n* = 8), and CRP-CDs treatment group (*n* = 9 each; 5.00 and 1.25 mg/kg = high dose and low dose, respectively). Model and CRP-CDs group mice were fed a high-fat diet (HFD), composed of casein, methionine, sucrose, corn starch, dextrin, anhydrous cream, cholesterol, cellulose, mineral mixture, vitamin mixture, and antioxidants (TP26304, TROPHIC Animal Feed High-tech Co. Ltd., Nantong, China), ad libitum for 16 consecutive weeks to induce MASH. From the 12th week, the CRP-CDs treatment group mice received daily intragastric administration of CRP-CDs for 4 weeks, while the normal control and MASH model group mice were given an equal volume of water. At the end of week 16, all mice were euthanized. Serum was obtained by centrifuging retro-orbitally collected blood samples at 3500 rpm for 10 min at 4 °C. Liver tissues were collected and frozen in liquid nitrogen. The samples were stored at −80 °C for subsequent analyses.

### 4.6. Measurement of Biochemical Indices

The levels of alanine aminotransferase (ALT), aspartate aminotransferase (AST), TG, TC, high-density lipoprotein (HDL), and low-density lipoprotein cholesterol (LDL) in the serum were measured using an automatic biochemical analyzer (Chemray 240, Shenzhen, China).

### 4.7. Histopathological Assessments

The liver tissues were washed twice in ice-cold saline, fixed in 10% (*V*/*V*) neutral buffered formalin, and paraffin sections were then cut to a thickness of 5 μm and stained with hematoxylin and eosin (H & E) for histological evaluation according to standard procedures. Oil Red O staining was performed on frozen sections, as paraffin processing dissolves neutral lipids. Tissues were OCT-embedded and cryosectioned. For quantitative analysis, the lipid droplet area fraction from Oil Red O staining was measured using ImageJ software.

### 4.8. Detection of Inflammatory and Oxidative Stress Markers in Liver Tissues

Liver tissue samples were processed for detecting inflammatory factors and oxidative stress indicators. Briefly, fresh liver tissues were excised, rinsed with pre-cooled PBS (pH = 7.4) to remove residual blood, and blotted dry. A 0.2 g portion was accurately weighed, minced on ice, and mixed with 1.8 mL cold PBS (*v*/*w* = 9:1). The mixture was homogenized on ice using a high-speed tissue homogenizer (Selt Biotechnology Co., Ltd., Shenzhen, China) at 12,000 rpm for 60 s × 2 cycles to obtain a uniform homogenate, which was centrifuged at 12,000 rpm at 4 °C for 15 min to remove debris. The supernatant was collected and stored at −80 °C. Then, the levels of tumor necrosis factor-α (TNF-α), interleukin-1β (IL-1β), and interleukin-6 (IL-6) in liver supernatant were determined using commercial ELISA kits (E-EL-M3063, E-EL-M0037, E-EL-M0044, Elabscience) following the manufacturer’s protocols. The levels of oxidative stress indicators, including malondialdehyde (MDA), superoxide dismutase (SOD), glutathione (GSH), and catalase (CAT), were determined using commercial assay kits from Nanjing Jiancheng Bioengineering Institute (Nanjing, China) following the manufacturer’s protocols.

### 4.9. Proteome Sample Preparation

Liver tissue samples (control, *n* = 6; model, *n* = 8; CDs-H, 5.00 mg/kg, *n* = 9) were collected and thoroughly washed twice with phosphate-buffered saline (PBS) to remove residual blood and cellular debris. Then, samples were lysed in ice-cold lysis buffer (4% SDS, 100 mM Tris-HCl, pH 8.0) supplemented with protease inhibitors for 30 min, followed by homogenization on ice (total 2 min, with 60 s bursts). The lysates were centrifuged at 16,000× *g* for 30 min at 4 °C, and the supernatants were collected for protein concentration determination using the BCA assay. Extracted proteins (100 ug protein) were reduced with 1 M dithiothreitol (DTT) for 30 min and alkylated with 200 mM iodoacetamide (IAA) for 30 min at room temperature in the dark. Proteins were precipitated with 10% (*w*/*v*) trichloroacetic acid (TCA) at 4 °C for 30 min, pelleted by centrifugation at 16,000× *g* for 15 min, and washed twice with cold acetone to remove residual TCA. After brief air-drying, the protein pellets were resuspended and digested with trypsin at an enzyme-to-substrate ratio of 1:50 at 37 °C for 16 h. The resulting peptides were desalted using SepPak C18 cartridges (Waters, Milford, MA, USA), concentrated by vacuum centrifugation (SpeedVac, ThermoFisher Scientific, Waltham, MA, USA), and analyzed by liquid chromatography–tandem mass spectrometry (LC-MS/MS, ThermoFisher Scientific, Waltham, MA, USA).

### 4.10. LC-MS/MS Acquisition

Samples were analyzed on an Orbitrap Exploris 480 mass spectrometer (ThermoFisher Scientific, Waltham, MA, USA) coupled to an UltiMate 3000 RSLCnano System (Thermo Fisher Scientific, Waltham, MA, USA) operating in data-independent acquisition (DIA) mode. Dried peptides were redissolved in solvent A (0.1% formic acid) and loaded onto a trap column (Acclaim™ PepMap™ 100 × 75 μm × 2 cm column, nanoViper 2kv C18, 3 μm, 100 A, Thermo Fisher Scientific) and separated on an analytical column (Acclaim™ PepMap™ RSLC C18 75 μm × 25 cm column, Thermo Fisher Scientific). Samples were analyzed on a single-column setup with an Aurora Ultimate analytical column (25 cm × 75 µm, C18 1.7 µm) maintained at 50 °C with a gradient of 3–90% mobile solvent B (80% acetonitrile with 0.1% formic acid) at a flow rate of 300 nL/min for 120 min. Instrument parameters were: RF lens frequency of 40%; ion source transfer capillary voltage of 25kV; and ion transfer tube temperature of 320 °C. Full MS scans were acquired from 350 to 1200 *m*/*z* in the Orbitrap mass analyzer at a high resolution of 120,000 with an automatic gain control (AGC) target of 3 × 10^6^ and a maximal ion injection time of 50 ms. MS2 scans were acquired at a resolution of 30,000, with an AGC target of 2 × 10^6^ and a maximum ion injection time of 50 ms. Higher-energy collisional dissociation (HCD) fragmentation was performed using stepped normalized collision energies (25%, 30%, and 35%), and data were recorded in profile mode. The DIA isolation scheme (centered isolation window) was: 361.5–517.0 *m*/*z*, 524.0–657.0 *m*/*z*, 664–797 *m*/*z*, and 807.5~1181.5 *m*/*z* (wide window). Mass spectrometry data were acquired using Xcalibur v4.4.16.14 (Thermo Fisher Scientific).

### 4.11. Proteome Data Analysis

Proteomic data from DIA mass spectrometry were analyzed using DIA-NN (v2.0, academic version). The Mus musculus reference UniProt FASTA (release 2024) was used as the search database. A library-free workflow was adopted with trypsin as the digestive enzyme (maximum 2 missed cleavages). Precursor and fragment ion *m*/*z* ranges were set to 400–1200 and 100–1800, respectively. Neural network scoring, interference correction, cross-run retention time alignment, and global normalization were enabled during data processing. False discovery rate (FDR) was controlled at 1% for both peptides and proteins. Qualitative identification results and quantitative expression matrices of proteins/peptides were exported for subsequent bioinformatics analysis.

Samples with protein-group counts outside the whiskers of the boxplot (statistical outliers) were excluded from downstream analysis. Protein abundance values were log_2_-transformed, followed by median normalization (normalizeMedianAbs Values from the limma package) to generate the final normalized protein expression matrix for subsequent analyses. All data processing and statistical analyses were performed using R software (v4.5.1). The analyses were performed using GSEA v4.4.0 downloaded from https://www.gsea-msigdb.org/gsea/downloads.jsp (accessed on 6 October 2025). The enrichment analysis was based on Biological Process GO and hallmark datasets, version v2025.1, accessed on 6th of October 2025.

### 4.12. Acute In Vivo Toxicity Study Evaluating the Safety of CRP-CDs

Specific pathogen-free (SPF) Kunming (KM) mice weighing 22 ± 1.0 g were purchased from the Laboratory Animal Center, Si Beifu (Beijing, China), and were provided with a Laboratory Animal Certificate of Conformity. The mice were housed in an environmentally controlled breeding room under conditions of 24.0 ± 1.0 °C, relative humidity of 55–65%, and a 12 h light/12 h dark cycle. All animals were acclimatized for 7 days prior to the experiment, with free access to standard rodent chow and water. These mice were randomly divided into control (*n* = 10) and CRP-CD (*n* = 10, 20.85 mg/kg, intraperitoneal injection [i.p.]) groups and were sacrificed 7 days after administration. Then, 20 μL blood samples were collected into EDTA anticoagulant tubes and then analyzed for routine blood indicators using an automatic hematology analyzer (Sysmex XI-800, Sysmex Corporation, Kobe, Japan), which mainly included white blood cell count (WBC), red blood cell count (RBC), hemoglobin (HGB), hematocrit (HCT), platelet count (PLT), neutrophil percentage (NEUT%), and lymphocyte percentage (LYMPH%). The remaining whole blood was placed in non-anticoagulant tubes and left at room temperature for 30 min to allow clotting. After centrifugation at 3500 rpm for 10 min at 4 °C, the serum was separated and analyzed using an automatic biochemical analyzer (AU480, Beckman Coulter, Inc., Brea, CA, USA) for AST, ALT, TC, TG, alkaline phosphatase (ALP), blood urea nitrogen (BUN), and creatinine (Cre). Mouse major organs were harvested, fixed in 4% neutral buffered formalin, routinely processed into paraffin, sectioned at 4 μm, and stained with H & E for morphological comparison between the two groups. All experimental procedures were conducted in accordance with the Guide for the Care and Use of Laboratory Animals and were approved by the Animal Ethics Committee of Shenzhen PKU-HKUST Medical Center.

### 4.13. Statistical Analysis

All statistical analyses were performed using IBM SPSS Statistics 20 and R software. The normality of data distributions was examined by the Shapiro–Wilk test and histogram plots. Levene’s test was used to assess variance homogeneity. One-way ANOVA was used for comparisons among three or more groups. Pairwise multiple-comparison analyses were performed using Tamhane’s T2 test when variance homogeneity was violated. A *p*-value < 0.05 was considered statistically significant. Data visualization was conducted with GraphPad Prism version 8.0.1 (La Jolla, CA, USA).

## 5. Conclusions

In the study, CRP-CDs were prepared using a green carbonization method, and they possess favorable biocompatibility and alleviate HFD-induced MASH. Proteomic analysis revealed that CRP-CDs do not act on a single target. Mitochondrial energy metabolism constitutes the core regulatory axis underlying CRP-CDs-mediated protection. This agent may coordinately balance lipogenic, antioxidant, and inflammatory signals, reversing multifaceted pathological dysfunctions in MASH. The findings are expected to provide a novel, safe, and effective therapeutic strategy for MASH. Still, our work is restricted to cellular and preclinical animal models. Further investigations focusing on long-term biosafety and in-depth validation of mitochondrial-related mechanisms are required to advance the translational potential of herb-derived carbon dots.

## Data Availability

Research data from the study are available from the corresponding authors upon request.

## Supplementary Materials

The following supporting information can be downloaded at https://www.mdpi.com/article/10.3390/molecules31213888/s1. Figure S1: Zeta potential of CRP-CDs. Figure S2: Quantification of Oil Red O-positive area fraction showing the percentage of lipid droplets within the liver tissue ROI. The data represent mean ± SD; * *p* < 0.05 and ** *p* < 0.01 as compared to the model group (model: FFA-induced untreated group; CDs-H: High; M: Medium; L: Low. H, M, and L= treatment with CRP-CDs at 125, 62.5 and 31.25 μg/mL, respectively). Table S1: A total of 1051 differentially expressed proteins (DEPs) were identified between the model group and the control group. Table S2: 91 DEPs were identified in the CRP-CDs-treated group compared with the model group. Table S3: Venn diagram analysis was used to assess the overlap of DEPs from two pairwise comparisons, identifying 67 common DEPs shared between the two groups.
