# Supplementary material for: Carbon Dots Derived from Carbonized Citri Reticulatae Pericarpium (Chenpi) Alleviate Metabolic Dysfunction-Associated Steatohepatitis
Source: Molecules. 2026 Oct 28;31(21):3888. doi: 10.3390/molecules31213888 (PMC13764711; doi:10.3390/molecules31213888)
Supplement: Supplementary file 1 [file molecules-31-03888-s001.zip › molecules-4558373-supplementary.pdf]

molecules

MDPI

Supplement Figure

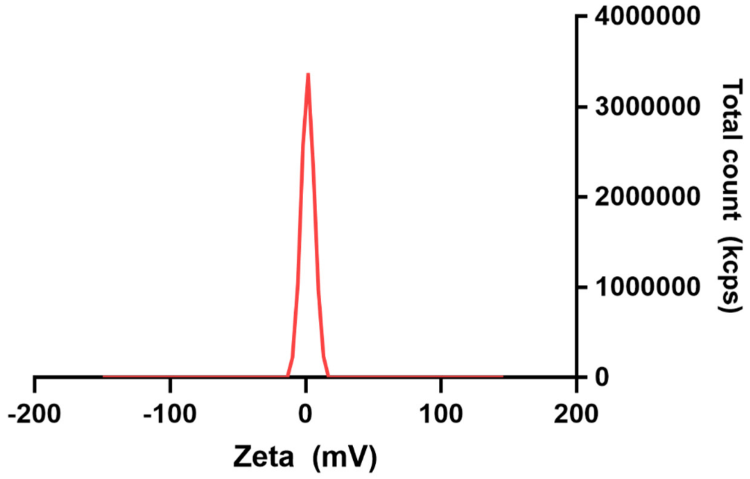

Figure S1. Zeta potential of CRP-CDs

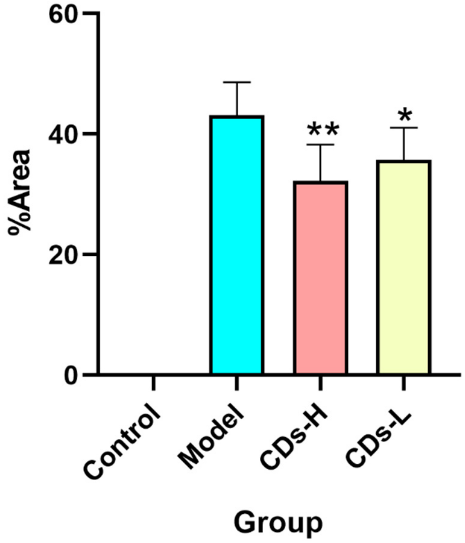

Figure S2. Quantification of Oil Red O-positive area fraction showing the percentage of lipid droplets within liver tissue ROI. The data represent mean ± SD; *p < 0.05 and **p < 0.01 as compared to the model group (model: FFA-induced untreated group; CDs-H: High; M: Medium; L: Low. H, M, and L= treatment with CRP-CDs at 125, 62.5 and 31.25 μg/mL, respectively)
